# Psychological Quarterly Retrospect

**Published:** 1859-10-01

**Authors:** 


### *Psychological Quarterly Retrospect.*

The practical psychologist will find much matter of interest in several events which have happened during the past quarter. The occurrence in the north of Ireland of an outbreak of religious enthusiasm, accompanied by certain physical and ecstatic phenomena, commends itself particularly to his attention. In other pages we have noticed briefly the nature of the physical phenomena which have been manifested in many individuals who have been influenced by the Irish "revival;" but as yet our information is too meagre to admit of a full consideration of the psychological aspect of the movement. We would, however, in the name of science, if from no other consideration, protest against a notion that has been too thoughtlessly emitted, that the whole phenomena of the revivals are to be regarded as the results of a mental malady. If intensified religious emotion of itself is to be regarded as a symptom of mental disease, why not intensified vicious emotion? Upon what ground is it assumed that highly excited religious emotion will not follow the same physiological laws as exaggerated emotion from any other source? The broad facts of the "revival" point to a heightened religious sentiment, increased by sympathy, in the districts within which the "revival" prevails; and among other and as occasional results of powerful and protracted, self-contained emotion, we find certain familiar, hysterical, and ecstatic phenomena, particularly among young people. These phenomena are mainly explicable upon physiological grounds; and except in so far as they may be misinterpreted and misused by enthusiastic professors of religion, they cannot be legitimately paraded as arguments either for or against the purity of the "revival." The same remarks are applicable also to the instances of insanity which are reported to have occurred during the progress of the "revival." In these exceptional phenomena we see the indications of emotion which has overleaped its normal bounds, which in the present instance has in too many instances been misunderstood, and which, if not checked, may lead to a plentiful crop of visionaries, ecstatics, and so-called demoniacal possessions,* as in

* According to a letter of the *Times*' Correspondent at the seat of the revival, written since the foregoing was penned, the movement has already produced a plentiful crop of visionaries. He writes :—

". . . There has been witnessed in this ultra-Protestant and enlightened community a series of visionaries, wonderful sleeps and trances, deafness and dumbness, spiritually induced, and, worst of all, cases of evident but clumsy imitation of several of the more ardent outbreaks of religious enthusiasm in the past century. It is the tendency towards such a result which has to be dreaded in Ireland, and which has to be guarded against. For history and experience alike teach us how apt the healthy progress of religious feeling is to be disturbed and perverted by those who see visions and dream dreams; how rapidly such characters multiply when once developed; and with how much greater avidity the multitude will listen to the fantastic revelations of a morbidly excited intellect, than to the more sober teachings of the Gospel. Already we learn that, in some instances, the visions and trance-like attacks of hysterical girls have been held up as manifestations of the Holy Spirit by one party, and evident signs of the diabolical character of the whole movement by another! Surely, it might be said to the former advocates, a more probable and natural explanation being open to them, that Christianity needs no extraneous assistance of this kind; and to the other, that it is best to keep Satan in the background as a primary causative agent in an upheaving of feeling, which, if the accounts we possess be true (and the statements that have been made to us in confirmation hardly permit a doubt respecting them), is certainly characterized by more marvellous examples of moral improvement, in several districts of Ireland, than have been witnessed in Europe for many years.

A clear recognition of the abnormal character of trance-like and convulsive seizures, by the ministers conducting the revival, and firmly setting the face against manifestations of exaggerated excitement, would soon eliminate this accidental and perturbing influence from the movement.

It is worthy of note that an outbreak of religious enthusiasm has occurred in Scotland, Wales, and in the North of England, but without ecstatic phenomena accompanying it.

So clearly does physiology teach us the influence of emotion upon man, that we venture to make a prediction that before long we shall witness a "revival" among the religious bodies in London. Those who have watched the influence of the great religious services to the working classes, held in Westminster Abbey, St. Paul's, and the principal music halls; who have noted the effects produced by the sermons preached by the many eminent ministers who have conducted the services; and who have marked the active exertions which have been made, particularly of late, to push religion into the innermost recesses the grossest kind of Romish imposture, pretended miracles; sacred names and words were marked on the bodies of women, in the manner of the *stigmata* often heard of in Catholic countries. The 'marks' said to have been made by the Spirit have been exhibited for money, and some of the filthy alleys and courts of Belfast have just reproduced scenes rivalling the imposture of the Cock-lane ghost."—(*Times*, Sept. 23.)

of the metropolis, cannot but be aware that a gradual heightening of religious sentiment has been taking place—that the *tension* of religious feeling has been increasing. When this culminates (and we have little doubt that it will, and the culmination may be brought about very quickly by the increased fervour produced by sympathy) we shall witness in London what the religious world calls a "revival." Indeed it would be a poor compliment to the zeal of those ministers of different denominations who for some time, in the special religious services referred to, have been preaching with renewed energy no vapid system of morality, but an active personal religion, to suppose otherwise. And truly there is not such a surplusage of godliness in the world that we can afford to quarrel with it if it comes upon us suddenly, in an old-fashioned, well-authenticated manner—with the sound as of "a mighty rushing wind."

Our clerical friends will doubtless think that we have taken a very grovelling view of this subject, but we keep to our functions, and seeking to remove a stumbling-block out of their way (over which we fear some have already tripped, if not fallen), and not cast one in it, we would caution them in the event of any religious enthusiasm becoming manifest, or existing among their congregations, to check at the very outset, as may be done by kindly firmness, all those abnormal manifestations of emotion—all tendencies to trance, ecstasy, visions, and convulsions—which are commonly characterized as hysterical, and which would, in the majority of instances, prove in the end most injurious to morality and religion.

From religious enthusiasm to murder and suicide may be a startling, yet for our Retrospect it is a necessary step. We cannot, however, avoid a passing allusion to the curious and interesting study of the intellectual status of the working-class, afforded by the present "strike" among masons, carpenters, and others engaged in the building trades of the metropolis.

The quarter has been surcharged with records of murder, some of which, from the confessions of the murderers, are of considerable interest as illustrating the etiology of crime; and others derive their chief value from the mode in which they bear upon the question of criminal responsibility. The instance worthy of most attention in the latter respect is one that was brought to trial before Mr. Baron Bramwell, at the Winchester Summer Assizes. The following is the *Times*' report of the case:—

Henry Benjamin Haynes was indicted for the wilful murder of Mary M'Gowan, at Aldershott.

The prisoner, when called upon to plead, said he did not wish to plead, he wished to be tried.

The judge told him he must plead "Guilty," or "Not Guilty."

The prisoner, after some time, pleaded "Not Guilty."

Mr. H. T. Cole was counsel for the prosecution; and Mr. Edwards defended the prisoner.

It appeared that the prisoner was a private in the 9th Regiment of Foot, stationed at Aldershott. On the 5th of March last, the prisoner and a comrade named Callender were confined to the barracks, but about 10 o'clock in the evening they broke out of barracks, and went to a public-house called the London Tavern, where women of low character resorted; they there met several girls. Callender selected one named Emma Turner, and the prisoner took the deceased, and they went upstairs to two different rooms, they remained there some time, when Turner left Callender, and went into the room where the prisoner and the deceased were. Turner told them it was time to get up; the prisoner left the bed and went into another room for a minute and returned; the deceased was then washing her arms; the prisoner went up to her, put his arm round her neck, and kissed her. The deceased then went into another room and the prisoner followed her, and in less than a minute, Turner and Davis, another girl who had come upstairs, heard the cry of "Murder!" They ran into the room and saw the prisoner and deceased standing together, but she was apparently sinking on the ground; the prisoner was taking his hand from her neck, and in that hand was a razor. They then observed that she was bleeding profusely from a wound in her throat. The prisoner walked to the table, put the razor in a case, and walked out of the room. The deceased said, "Lord, have mercy on my soul; I hope my poor mother will forgive me, I have been a wicked sinner," and died in less than five minutes, in consequence of the wound in her throat. The prisoner was taken into custody. He said his mind had been uneasy ever since he had left America, as he had seduced a young woman there, and she had a child by him, and he left her. He was asked what had made him kill the deceased. "I don't know; poor girl, she never did me any harm. It was not her I intended to kill, it was Margaret Cheltenham, who caused me to be kept in the hospital, and it was the devil did it." Margaret Cheltenham lived in the adjoining house.

Callender stated in cross-examination that he had come with the prisoner from America. He appeared to have something on his mind, and was not like what he had been before he went. He seemed at times hardly to know what he was doing. He went about his ordinary duties.

Mr. Edwards addressed the jury on behalf of the prisoner. He urged them to dismiss from their minds everything they had before heard or read. If the simple question was whether the prisoner had committed this act, he should not address them or take up their time; but he must ask them to consider whether the prisoner's state of mind at the moment was of such a character that he was not conscious of what he was doing. It was one of the most extraordinary cases he had ever heard; it was involved in the greatest mystery. People did not commit murder without some motive or reason; if they did so, it was a proof that their mind was not in its proper state—it was disordered. Could they conceive a person in his sane mind without any motive committing such an act? It might be a dangerous doctrine; but at the same time it was a true one in an instance like the present. If there was not this state of insanity, then there must have been provocation, so as to reduce the offence to that of manslaughter.

Sergeant Herman.—I have known the prisoner four years. I was with him in America. After his return I observed a great alteration and peculiarity in him. When he took a drop of drink he appeared rambling in his mind; and also at other times, very often. He was a tailor, and therefore did not do much duty.

Cross-examined.—He used to be talking about a woman in America. He seemed sorry that he could not marry her. He said, "Oh, my head!" He appeared quite out of spirits. One time he had drink he went away to York and came back very honourably, and gave himself up. He used to say, "Oh, my head! that poor girl!" He did not appear to know what he was doing. While he was a prisoner for desertion, if he was told to do a thing, he would do quite the contrary. He bore an excellent character in the regiment.

Mr. Cole replied, observing that it would be dangerous indeed if a prisoner were to be relieved from his responsibility upon such evidence as that which had been produced for the prisoner in this case. It was frequently impossible to discover or fathom a motive. Was there anything to show that the prisoner did not know the act he was committing? As to any provocation, there had not been time for it.

Mr. Cole then said that for the satisfaction of his lordship he had sent for the surgeon of the gaol, who was in attendance and ready to give evidence.

The judge doubted whether, after the speeches, it would be right to put the witness in the box, but he would consult Mr. Justice Crompton.

Mr. Baron Bramwell, upon his return, said his learned brother agreed with him that it was better to be regular, and, therefore, he should decline to have this gentleman examined.

The learned judge then proceeded to sum up the case. There could be no doubt that the prisoner had killed the deceased; it had been proved over and over again; that being so, he was guilty of murder, unless some cause was shown to reduce the crime, or to show that the prisoner was not responsible for his act. There was no evidence of any quarrel or provocation so as to reduce the offence to manslaughter. The only question, then, really was as to the state of mind of the prisoner at the time he committed the act, and he should not flinch from the discharge of his duty, and he trusted the jury would not, for he thought that although it might be a painful thing to find the prisoner guilty, yet it would be equally painful—it would be a most painful thing for a jury to consider that they had violated their oaths. The learned judge then stated to the jury what the law was, and read to them the opinions given by the judges upon this point. As to the question of malice, he said malice might be either expressed or implied, and if a deliberate act was committed by one person against another, the law would imply malice. It would be a most dangerous doctrine to say that because you could not show a motive a man was to be acquitted. The question was whether this prisoner had a sufficient degree of reason to know that the act was wrong. If he knew what the act was, and that the act was wrong, the prisoner was punishable for the act. When a man committed murder, the influence of religion, the law, and humanity had been overcome, but that was not to relieve him from punishment. Did the woman die by the hand of the prisoner? If so, he was guilty, unless he did not know the nature of the act, or did not know that it was wrong. Those were the only two matters for their consideration. It was to the advantage of all to obey the law, and every improper acquittal was detrimental to the interests of society.

The jury retired for upwards of two hours. They then sent a note to the judge to state that some of the jury had doubts as to the state of mind of the prisoner, and therefore requested some further explanation of the law as regarded insanity.

The judge directed the jury to be sent for, and on their coming into court asked them if they had any observation to make.

One of the jurors said the prisoner seemed to have acted under an uncontrollable impulse.

The judge said that did not make the offence the less mnrder. Malice was implied when there was a deliberate cruel act committed, however sudden it might be. It was no matter how sudden the impulse—whether it was the result of long previous deliberation, or whether it was the impulse of an instant —it would be as much murder in one case as in the other. No jury could properly acquit on the ground of insanity if they believed the accused was conscious of the act he was committing, and that he knew that act was contrary to law. If they gave a verdict contrary to this, the result would be to increase the number of cases of uncontrollable impulse. The object of the law was to prevent an impulse from being uncontrollable. If the man was conscious of what he was doing, and that it was contrary to law, he was punishable.

The jury, after putting their heads together for a minute, returned a verdict of *Guilty*.

The judge, having put on the black cap, addressed the prisoner: I don't think that the jury could properly have given any other verdict. No doubt you killed that young woman, and there is no doubt that you were in a state of mind which would not have justified an acquittal. My duty is a short one. I don't desire to reproach you, or to give you pain, because I can't help feeling sorry for you. You bore a good character, and you had a sort of regret for what you had done abroad, which makes one sorry for you; but, without saying anything to give you pain, it is my duty to warn you that the sentence will be carried into effect, and it is my duty to tell you that it was a very cruel and a very bad act, for you went out with a determination to do some mischief, and this unfortunate creature came in your way, and you took her life, and you cannot expect your own to be spared. Therefore, make your arrangements and preparations, and you will have all the advice you may require as to what you ought to do—it is not my duty to enforce it upon you—my duty is to pass the sentence of the law. His lordship then passed the awful sentence of the law on the prisoner, who was then removed from the dock.

The judge omitted the usual concluding prayer.

Soon after the trial, the murderer was respited—a result which confirms the conclusion to be derived from the history of the case, that, although the summing-up of the judge and the verdict of the jury might satisfy the strict requirements of the law, they did not satisfy those of justice. The evidence adduced in favour of the prisoner, scanty though it was, clearly indicated the existence of mental derangement immediately prior to the deed, and the observation he made after committing it was exceedingly significant. When asked what had made him kill the deceased, he answered: "I don't know; poor girl, she never did me any harm. *It was not her I intended to kill, it was Margaret Cheltenham*, who caused me to be kept in the hospital, and it was the devil did it." This remark would make it highly probable that the act was performed under the direct influence of a delusion, which, if it had been proved, would have established the man's irresponsibility strictly within the law. But no medical evidence was produced, and the man was left to take his chance upon the general evidence. This, however, as we have already remarked, was so strongly indicative of mental derangement, that the jury, after long deliberation, came back into court to ask guidance from the judge as to the doubt which beset their minds respecting the criminal's sanity.

A juror thought that the prisoner had acted under an uncontrollable impulse; but to this the judge replied, that supposing such were the case, it would not make the offence less murder; and he then proceeded to speak, not of an uncontrollable impulse—the question put—but of an *unrestrained* impulse, confounding the uncontrollable impulse of insanity with the unrestrained impulses of vice! His lordship next restricted the legal irresponsibility of insanity to those cases in which the lunatic was not conscious of the act he was committing, and of its being contrary to law—a dictum the insufficiency of which, to deal with the *facts* of homicidal insanity, has been again and again felt on the bench, and the use of which has again and again rendered it necessary, in order to avoid unjust consequences, to have recourse to expedients similar to those used in this case. As a species of apology for, or justification of the dictum, the judge added,—"If they (the jury) gave a verdict contrary to this, the result would be to increase the number of cases of uncontrollable impulse." Here, again, we presume the judge meant unrestrained for uncontrollable. Well, a verdict of "Guilty" is given, the murderer is sentenced to death, and in another week he is respited! And why? If the man were sane when he committed the deed, he was guilty of as bloody and deliberate a murder as the annals of crime can show; if he were insane, why should the extreme sentence of the law be turned into a meaningless exhibition? Which course of action is most likely to beget an increased number of pleas of uncontrollable impulse, that which slurs over the plea, or attempts to crush it beneath an arbitrary legal dictum, and which, when unjust, has to be systematically remedied by an appeal to the Crown for mercy, or if not unjust, leaves the subject liable to be seized upon at any moment by a capricious philanthropical sentiment, and paraded as an injustice? Or, that course which thoroughly and effectively analyses the plea in a public court, which clearly exposes its fallacy, if it be fallacious, *in respect to the case tried*, and not deductively from an imperfect rule, or stamps with certainty its correctness if right; and which, having so done, records a conclusive and indisputable sentence: or, any doubt being present, that doubt is recorded in open court, for the prisoner's benefit, and pursued to its consequences by the judge himself, and not left to the public? We have little doubt that the public would unhesitatingly pronounce the former course to be the most dangerous to law and morality.

A case somewhat similar to that of Haynes's, but which has not yet come to trial, occurred near Gloucester, on the 31st of October last. The following report is from the *Daily Telegraph* (Sept. 1)—

"A fearful tragedy was enacted yesterday morning at the town of Lydney, on the South Wales Railway, between this city and Chepstow. It appears that an elderly gentleman of the name of Pownall, a retired physician, and a native of Wroughton, Wilts, has been residing with Mr. Leete, surgeon, of Lydney, for the last three weeks. He had formerly been an inmate of Northwoods Asylum, near Bristol, but had recently been discharged as cured, with a certificate to that effect from Dr. Davey, the medical superintendent. During his residence at Lydney he had been quiet and gentlemanly in his habits and conduct, and has never shown any symptoms of unsoundness of mind. About six o'clock yesterday morning he knocked at the servants' bedroom door, and called them up. One of them—a girl named Louisa Cooke, aged fifteen years—dressed herself quickly and went out. In a minute or two, the other girl, who had not risen, heard a scuffle on the stairs, and a cry of 'Murder.' She got up and looked out, but could see nothing; and having called out, 'What is the matter?' received no answer. She returned into the room to dress herself, leaving the door partly open, and in a minute or two Dr. Pownall, in his shirt sleeves, looked into the room and said, 'Make haste, get assistance, some one has murdered her;' and then retired to his own room, locking the door. Sergeant Pope, of the police, was immediately called in, and on going to Mr. Leete's bedroom he found the poor girl Cooke lying dead upon the floor, with a fearful wound in her throat, from which a large quantity of blood had flowed. The poor girl had rushed into the room, exclaiming, 'Master, he has murdered me; I must die;' having repeated which two or three times, she fell down dead. The policeman immediately proceeded to Dr. Pownall's bedroom, which was locked; and as he could get no reply on requesting that the door might be opened, he burst it open. He found Dr. Pownall sitting on the bed, partly undressed; his shirt was spotted with blood, and lying on the dressing-table was a razor, with blood on the blade and handle. Pope apprehended him on the charge of murdering the girl, to which Dr. Pownall made no reply, but quietly dressed himself and proceeded to the police station. Information was immediately conveyed to James Teague, Esq., the county coroner, who issued his warrant for an inquest, which was held last night at the Feathers Hotel, Lydney, the Rev. H. Philpot, the vicar, acting as foreman of the jury.

"Mary Ann Fryer, fellow-servant of the deceased, and the policeman Pope having deposed to the above facts,

"Dr. Pownall, who had not hitherto spoken, but sat at the table shading his eyes with his hands, said slowly, and in a deep voice, 'I can tell you; I unfortunately did it. I can hardly assign any motive. I felt I was bound to do something, and I could not resist it.'

"Mr. Charles Lydiat Leete deposed: I am a surgeon, residing at Lydney, and deceased was in my service. This morning she rushed into my bedroom with her throat cut, and died in a few minutes. When she came in, she exclaimed, 'Master, he has murdered me; I must die.' I have known Dr. Pownall for the last three weeks. He has been residing with me that time. He has all along appeared sane and reasonable, and I saw no change in him when I parted from him last night. He showed me a certificate from Dr. Davey, which certified that he left his lunatic asylum cured.

"The coroner having summed up, the jury immediately returned a verdict of wilful murder, and Dr. Pownall was committed for trial at the next assizes. He was removed to Gloucester County Prison this morning.

"The unfortunate girl is the only child of her parents, who are poor persons residing near Berkeley."

There can be no doubt that this is an instance of homicidal mania, and hardly any doubt that Haynes's case belongs to the same category.

Let us contrast these two examples of murder committed by lunatics with two committed by sane men, as recorded by themselves.

Matthew Francis, aged twenty-six years, tailor and hawker, was charged before Mr. Justice Willes, at the Monmouth Assizes, on the 6th August, with the wilful murder of his wife, Sarah Francis, on the 12th March, 1859, at Newport. The following statement, made by the prisoner before the magistrates, was put in as evidence against him:—

"Last Monday fortnight I got up and lit the fire. I got the breakfast ready against my wife came downstairs. I had a bloater roasting by the fire. Something was not right about the bloater, and she took it and flung it at me. I asked her what she did that for, and she told me to ask her ——, &c. I said that was a very pretty expression for a woman to make use of. She told me it was good enough for such a bloody animal as I was. I told her not to say that or I would give her a smack on the head. She said, 'What, a —— —— son like you!' I told her I was no —— son. She said. 'If you ain't, you are a cow's drop.' She rose the fire shovel, and struck me on the shoulder. I ran to the door, and laughed at her. She said, 'Don't laugh at me, you —— thief.' She ran after me, got me by the hair of the head, and scratched my face. Mr. Richards came in, and asked what was the matter, and told my wife that she ought to be ashamed of herself to make use of such expressions. She ran at me again, and struck me. I struck her. She wanted to go out. I would not allow her. She told me she would put me in prison. I told her that was a foolish idea, as I could prove it was her fault. Then I let her go out into the passage. I went after her, and asked her to come back. She sat down in the passage. I pulled her, to pull her into the house again. I went into the house, and in about half-an-hour she came in. Mary Townsend came in. She made tea. She wanted to go out with Mary Townsend. I would not let her go. She took a knife from the table, and said she would cut my throat if I would not let her go out. Then I let her go. I watched where she went. I found her at Hawkins's. She said she would never rest her bones alongside me again. I saw her frequently, She refused to come back to me. On Friday evening I went to Hawkins's. She insulted me as soon as I went in. I told Mrs. Hawkins she ought to be ashamed for keeping her. I sent for some jugs of beer and eggs, and my wife made egg-flip for herself. I offered her my glass to drink out of. She would not take it out of my hand. She did after I had persuaded her. I asked her if she intended to live with me or not? She rose up a pint jug to throw at me, and said not to put such a question to her. She began cheeking me. I said a few words. I told her to come home, that my life was a misery to me, and that I would sooner go to the gallows than be separated from her. I told her to make up her mind by the morning, and then I left. On Saturday morning, at about nine o'clock, I went to her again. She told me to go out, that she did not want to see me. She cursed me. I told her not to put herself in a bad way. I sat down by her. I asked her to go home. She said she would not rest alongside of me. There was a knife on the table. I asked Mrs. Jones to lend it to me and I would kill her with it. I rubbed it along the pan on the table and I touched my wife on the back, and she said, 'Don't put your hands on me.' She called me many disgraceful things. I went out into the closet and sat down. I began to cry. Then, when I thought to myself about the razor I had in my pocket, I was not thinking of it before; I brought it out with the intention of having it ground. I took it out of my pocket and tied a piece of thread I had in my pocket round it. Then I went into the house again. I begged her to come home, and not to make a fool of herself and me too. I rose up and told her I was determined to kill her if she would not come home with me. She began to sing a song. I told her not to sing songs, there was more need for her to sing hymns. She laughed aloud when I said that. Then I caught her by the forehead, and said, 'Now, Sarah, will you come home or not?' She said, 'I will not.' I said, 'What! never come home?' She said, 'No; never!' Then I drawed the razor across her throat. She caught the razor on the palm of her hand. She let it go again, and caught on my wrist, and drew my hands down, and the razor caught on her cheek as she drew my hand. She heaved her two arms up and caught me round the neck, and pulled my face to her and kissed me. I told her 'I had murdered her, and was willing to die for her.' The last word she spoke was, 'Oh no! you shan't die.' I suppose she did not think it was done to the purpose. That is all.

"MATTHEW FRANCIS, his × mark."

The perpetrators of the brutal murder of Mr. Stephenson, at Sibsey, in Lancashire, on the 16th of March last, when lying under sentence of death in Lincoln Castle, made the subjoined confession to Mr. Rushton, a dissenting minister. Carey's statement was read to Pickett, and Pickett's to Carey; they were both pronounced to be correct, but they differ materially from the statements previously made by the murderers.

PICKETT'S STATEMENT.

"Lincoln Castle, 2nd Aug., 1859.

"On leaving the Ship Inn, at Sibsey, Northlands, on the 16th day of March, the night of the murder, between ten and eleven o'clock, the first word Carey said to me, after leaving the public-house was, 'We will go and rob old Stephenson.' I said to him, 'He will know us.' Carey said, 'No: I will stop him from that.' I was before Carey; we took two sticks from Mr. Teesdale's fence; I pulled one, and the other was picked up by Carey. We went to Mr. Coates' gate and altered our dresses, walked some distance, and laid down on the road-side, with the sticks beside us. The old man came down the road, crossed over to us, touched me over the head, and said, 'What are you doing here, my boys? I don't know you. Who are you? David, is it you?' I suppose he meant his grandson. Carey got on his knees, and pulled the old man down. Stephenson struck Carey with his walking-stick while falling. I held his head while Carey robbed him. I got up and struck him on the ground until my stick broke all to pieces. Carey got off when he had robbed him and beat him about the head; then carried him into the sewer. Carey went back across the road to fetch his stick, and struck the old man eight or nine times over the head until his stick broke all to pieces. Then the old man stood up in the sewer; Carey shoved at him with the broken part of his stick, trying to push him down into the water. Mr. Stephenson got from him, and walked to the other side of the sewer. Carey then threw the broken part of his stick into the sewer, and said to me, 'Go round to the other side and kill him.' I went round to Mr. Coates' yard, got a piece of a rail, and went to the old man. Mr. Stephenson had the thorn stick produced in court on his shoulder, and appeared to be going home. Carey said, 'Make haste, or he will get home.' I went behind him and struck him on the side of the head, and knocked him down; I hit him till my stick broke all to pieces (this was the rail or broad stick). I then took the stick Mr. Stephenson had, and hit him about the head till he was dead. I dragged him to the hedge; I was trying to throw him into the sewer, but could not. I told Carey I could not throw him over by myself, and he must come and help me. Carey said, 'Try again; you can get him over.' I then took hold of his legs, reared him up on end, and tumbled him over into the sewer. After that we both left, one on one side of the sewer, and the other on the other. We went on till we got to my father's seven-acre gate. Carey had the papers produced in court, and threw the old man's tobacco-box into the river opposite the gate. We went across the river in my father's boat, and went on till we got to my brother John's house. We were going to George Sands' hovel to sleep. Carey said, 'We had better go across brother John's garden, or the police will meet us.' We slept together in Sands' hovel till morning; got up about four o'clock. Carey said, 'We had better look if there be any blood on our clothes.' We could not find any, but I had some blood on my face, which Carey washed off. We laid down again till six o'clock. Mr. Sands came into the hovel to feed his beasts; he tumbled over my legs, and said, 'What are you doing here?' Carey said, 'We were locked out last night.' Mr. Sands asked Carey if he would sow him some onions. We got up and went to the Ship Inn; on the road Carey gave me the sovereign. Carey put a stone into the old man's bags, and threw them into Richardson's pit. We went round to Richardson's back door to see if they were up. I went home, and Carey went to his brother's. We did not think of robbing or doing anything else to Mr. Stephenson when we left the public-house. My father has often told that if I kept company with Carey I should be either transported or hung. I did not do as advised by my parents or friends. I was very much frightened the night it thundered and lightened; I thought Mr. Stephenson was coming to me in the cell. I am truly sorry for what I have done. The sentence passed upon me is a just one, and I deserve it. I feel for my parents, particularly my poor mother. I have no person to blame but myself. I felt relieved when the chaplain visited me on Thursday, after the sentence was passed; and I earnestly pray to God to forgive me for murdering the poor man. I believe I struck the blow which caused death. The reason I made the statement at Spilsby was to clear myself. If Carey had been present at the examination before the magistrates I should not have made that statement. The judge has done justice; if there was no justice there would be no living. I have been kindly treated during my confinement; I feel resigned to my fate, and hope to have forgiveness; I am striving for it as well as I can. I hid the money—one sovereign—which Carey gave me in the thatch of David Bank Richardson's furnace house, about 18 inches from the end wall on the west side, between the first and second spar; and his knife, that Carey had, is just outside the furnance at the corner by the chimney, which I should like to be given to Mr. Stephenson's son. We never had any handkerchiefs, and Carey's statement about them is false. I have nothing else to state, and the above is the truth.

" (Signed) WILLIAM PICKETT."

CAREY'S STATEMENT.

"Lincoln Castle, 2nd Aug., 1859.

"Oh dear Mr. Rushton, my position is an awful one; I hope I shall be forgiven: it is all drink. I am truly sorry I did not take your advice. Mr. Stephenson was always a friend to me; he took me in when I was turned out of my own house. I hope his friends will forgive me. I hope the old man's soul is in heaven. I feel reconciled, and the sentence passed is what I deserve. We was at Richardson's, the Ship Inn; I called William Pickett out of doors, and asked him if he would go with me to rob Mr. Stephenson. When we left the public-house we arranged to go into the house and say to the people that we should sleep in the boat (Pickett's father's boat). We stayed till between ten and eleven o'clock. The landlord asked if we thought anything about going home; it was bed-time. I asked him to put a quart of ale into a bottle, as we were going aboard. He put three gills into a porter bottle. We went over the river in Pickett's father's boat; we went on before Stephenson, got a stick each, and laid down in the lane where Mr. Stephenson had to go to his son's. He came up and said, 'Hallo, my lads, who are you? Get up and lay in some of these yards.' I think he did not know us. We both jumped up on our knees, and threw him backwards. Pickett held his head down till I got his money. I struck Mr. Stephenson, and then Pickett struck him while on the floor several times. Pickett took hold of his head, and I took hold of his feet, and threw him into the sewer. Mr. Stephenson got up on his feet while in the water, and we both struck him again. He turned to go across the sewer, and get out on the other side. I said to Pickett, 'Go round.' Pickett went round, got another stick, and struck him on the side of the head, knocking him down on the floor. I told Pickett to throw him into the sewer, which he did. I never went to the other side of the sewer. I know that I am the worst, and persuaded Pickett into it. We slept in Sands' hovel till half-past five o'clock. Sands found us. We left there. Pickett took a sovereign; I kept a knife and three shillings and sixpence. The two little bags were sunk in Richardson's pit; the pocket-knife I hid in the corner of Richardson's potato-house by the privy. I don't know what Pickett did with his money. It is false about having any handkerchiefs. That is all I have to say, and the above is the truth."

[This statement was witnessed by Mr. James Foster and one of the warders. Carey signed it with his mark.]

How remarkable the difference in the self-assigned motives for the perpetration of the deed in the two former and in the two latter instances of murder! Yet neither the motive which led Haynes, nor that which impelled Dr. Pownall to do murder, could be allowed for one moment as a plea for exemption from the consequences of the act, any more than the statements of Francis or of Pickett and Carey. What then constitutes the differential element in the cases justifying and calling for the exemption of any of the murderers from the extreme penalty of the law? Simply the existence, in the cases of Haynes and Pownall, prior to the act, of manifest indications of mental derangement, of insanity, and their probable connexion with the act, and the absence of any such symptoms in the other cases. Mental disease being unequivocally present in the instance of Haynes and Pownall immediately before the murder, and the anomalous motives assigned by them and the circumstances under which the act was performed, being, as experience (putting aside physiological reasoning) tells us, more consistent with the workings of the insane than the sane mind, justice demands that these most probably irresponsible agents should not be consigned to the gallows. Not that they should be acquitted and given back to society. The law, righteously and for public safety, retains in durance all persons acquitted of capital crimes on the ground of insanity. The question is solely one of hanging or not hanging an agent who may be irresponsible for his act by reason of mental disease. The law admits the plea, but the judges persist in dealing with it almost entirely by deduction from rule and precedent, neglecting the necessary adjunct of induction by fact and observation.

A curious confession of self-restraint gradually succumbing under the influence of continued domestic irritation, and of passion taking a murderous bias, occurred at the Westminster Police Office, on the 23rd August last. There was some similarity in the case of the applicant to the magistrate and that of Matthew Francis before he murdered his wife.

A powerfully-built labouring man solicited the magistrate's assistance and advice, when the following dialogue occurred:—Applicant: I want you to do something with my wife.—Mr. Arnold: What is the matter with her? Applicant: Why she robs my children, pawns everything I've got in the place, and gets drink with it; and if she don't reform, I shall have to do something with her that will bring me here. I shall do something to her that'll get me transported or something.—Mr. Arnold: I hope you will not be driven to do that. What do you want me to do for you? Applicant: I want you to separate us, and let one or the other of us take the children.—Mr. Arnold: I cannot assist you in that matter. Under the circumstances, the best thing you can do is to separate, you to allow her something per week, which, added to her industry, will keep her, but I have no power to interfere. The arrangement must be entirely between yourselves.—Applicant: Well, all I've got to say is, that as you will not interfere there will be murder committed very soon.—Mr. Arnold: I hope there will not be.—Applicant: Wont there? You will have me here before you before long for murdering her, and then I shall be hung, or something. I'll kill her if things do not alter.—Mr. Arnold: I think, after that, the safest thing I can do is to endeavour to prevent your resorting to violence. I shall order you to enter into your own recognizances in 20*l.* to keep the peace for six months. —Applicant, who for the moment was struck speechless with astonishment, entered into the required recognizances.—*Standard.*

An equally curious case *apropos* of suicide is also contained in the police records of the quarter.

A striking case occurred here on Wednesday last. It appears that a man named Francis Duncan, a shiprigger, belonging to Dundee, called at the shop of Mr. Burrell, chemist, about midday, and asked for some prussic acid for killing rats. He was informed by Mr. Burrell that the article was not usually applied to such a purpose, and was refused. Duncan then went to a second shop of Mr. Burrell, at the High-street Port, where he asked for oil of vitriol (sulphuric acid) from Mr. John Clark, the young man in attendance, who questioned him as to the intended use of it, and who, in answer to a question, informed his customer that it was poisonous, but that it was a very cruel and torturing method of killing vermin. This appeared to excite doubts in the man's mind, for immediately he inquired for prussic acid instead, and Mr. Clark retired for a minute and returned with a bottle duly *labelled* with the article required, but which meantime, he had filled with pure water and a few grains of Rochelle salts, to give it a flavour. A small bottle being filled therewith, the man asked if it would kill, and was informed that such a phial of acid would kill half-a-dozen people. Having dipped his finger in the liquid, and applied it to the tongue, he expressed himself satisfied, as he used it often to cure toothache; and on being cautioned as to its use he left the shop. Meanwhile information had been lodged at the police office of the theft of a silver watch from the house of Mrs. Reid, lodging-house keeper, Shore Wynd, the property of one of her lodgers, and it was suspected that Duncan, who had been lodging there, was the thief. The police, after an active search, had succeeded in recovering the watch in a pawnbroker's; and having their suspicions confirmed as to the guilty party, set out in his track, and succeeded in apprehending him. Just, however, before the officers seized him he drew forth the fatal phial, and instantly swallowed its contents, and then placidly informed the officers that he had taken poison, and would in a few minutes be beyond the reach of human law. The consternation ensuing was immediate, and a medical gentleman was promptly sent for, who had the satisfaction of finding emetics and other appliances, suggested by the probable nature of the supposed poison, followed in a few minutes by the revival of the poor man, who, under the impression that he was really dying, was no less astonished himself at being so easily rescued from otherwise inevitable death. He has since been examined and committed to gaol, and is none the worse for the dose. The good judgment and discretion shown by Mr. Clark in this instance deserve the highest commendation, as, unquestionably, had he not exercised the tact displayed, the poor man's life in all probability would have been sacrificed.—*Montrose Standard.*

We may mention also, as an example of one of the horrible eccentricities of suicide, an instance which occurred very lately, of an insane patient (a lady) who deliberately set fire to her clothing, and endured all the fierce scorching of the flames "without a shriek or cry of any sort." Death resulted, and it is stated that the patient had once before attempted to commit self-destruction in a similar manner.

The point of greatest interest, perhaps, in connexion with suicide, during the quarter, is a threatened collision between the Law and the Church on the question of the interment of self-murderers. The circumstances are thus stated by the *English Churchman*, July 28th :—

"In our Parliamentary Report, last week, we gave the substance of a petition presented by Lord Brougham, complaining that a clergyman in Leicestershire had refused to bury one of his parishioners who had committed suicide, while in a state of 'temporary insanity,' according to the verdict of the coroner's inquest. The Lord Chancellor (Lord Campbell) is reported to have stated that there could be no doubt whatever that the clergyman was wrong in point of law—that he had no right to form and act upon his own opinion contrary to that of the coroner's jury—and that the deceased was entitled to Christian burial according to the rites of the Church of England. In other words, we presume, the law of the matter is declared to be, that a clergyman must accept the verdict of the coroner's jury as decisive of the matter, and that unless that verdict be one of *felo de se,* the deceased is entitled to Christian burial, even though the evidence in the case, or the clergyman's own knowledge of the circumstances and character of the deceased, may convince him that the verdict was an untrue one.

"We must confess our ignorance of the statute or common law upon which the Lord Chancellor's opinion is founded, but we do know that when we turn to our prayer books we find it distinctly declared in the rubric prefixed to the burial service, that 'the office is not to be used for *any* that . . . . have laid violent hands upon themselves.' From this it would appear that the clergy have nothing whatever to do with the state of mind of those who have died by their own hands. Whether they were in their right senses or whether they were not—whether their insanity was temporary or permanent—whether they were responsible for their actions or whether they were not—all this is clearly beyond the scope of the rubric, which is confined to this single and simple question—Did the deceased lay violent hands upon himself—in other words, did he die by his own hands? If the clergy are bound by the letter of the rubric, we must be excused for hesitating to receive the *dictum* of even a Lord Chancellor, when it contradicts that rubric. For the same reason, we cannot implicitly receive the assertions of Dr. Burn—that 'the proper judges, whether persons who died by their own hands were out of their senses, are the coroner's jury. . . . . On acquittal of the crime of self-murder by the coroner's jury, the body in that case not being demanded by the law, it seemeth that a clergyman may and ought to admit that body to Christian burial.' In considering this subject we cannot lose sight of the fact that serious temporal penalties, including the forfeiture of property, may be legally enforced in cases where a verdict of self-murder has been returned; and that formerly this was followed by a degrading mode of burial, in unconsecrated ground. It is quite notorious that these circumstances have materially affected the traditions and habits of coroners' juries. This mode of burial having been abolished, and exchanged for burial by night, without service, but in consecrated ground—which is quite consistent with the rubric—it appears to us that so far as coroners' juries deal with the state of mind of the deceased, they do so in respect of the temporal penalty alone, and that their opinion upon that point is not an essential part of their verdict, and certainly cannot interfere with the plain and simple rubric of the Prayer Book, which points to no condition of the kind.

"There is much truth and common sense in the following observations by Wheatley. Speaking of the degrading mode of burying suicides which formerly prevailed, he says:—

"'This indignity, indeed, is to be only offered to those who lay violent hands upon themselves, whilst they are of sound sense and mind; for they who are deprived of reason and understanding cannot contract any guilt, and therefore it would be unreasonable to inflict upon them any penalty. But then it may be questioned, whether even these are not exempted from having this office said over them; since neither the rubric nor old Ecclesiastical laws make any exception in favour of those who may kill themselves in distraction, and since the office is in several parts of it improper for such a case. As to the coroner's warrant, I take that to be no more than a certificate that the body is not demanded by the law, and that therefore the relations may dispose of it as they please. For I cannot apprehend that a coroner is to determine the sense of a rubric, or to prescribe to the minister when Christian burial is to be used. The scandalous practice of them and their inquests, notwithstanding the strictness of their oath, in almost constantly returning every one they sit upon to be *non compos mentis* (though the very circumstances of their murdering themselves are frequently a proof of the soundness of their senses), sufficiently show how much their verdict is to be depended on. It is not very difficult indeed to account for this. We need only to be informed, that if a man be found *felo de se*, all he was possessed of devolves to the King, to be disposed of by the Lord Almoner, according to his discretion; and no fee being allowed out of this to the coroner, it is no wonder that the verdict is generally for the heirs, from whom a gratuity is seldom wanting. They plead, indeed, that it is hard to give away the subsistence of a family; but these gentlemen should remember that they are not sworn to be charitable, but to be just; that their business is to inquire, not what is convenient and proper to be done with that which is forfeited, but how the person came by his death; whether by another or himself; if by himself, whether he was *felo de se*, or *non compos mentis*. As the coroner indeed summons whom he pleases on the jury, and then delivers to them what charge he pleases, it is easy enough for him to influence their judgments, and to instil a general supposition, that a self-murderer must needs be mad, since no one would kill himself unless he were out of his senses. But the jury should consider that if the case were so, it would be to no purpose for the law to appoint so formal an inquiry. For, according to this supposition, such inquiry must be vain and impertinent, since the fact itself would be evidence sufficient. It is true, indeed, there may be a *moral* madness, *i.e.*, a misapplication of the understanding in all self-murderers; but this sort of madness does not come under the cognizance of a jury; the question with them being, not whether the understanding was *misapplied*, but whether there was *any* understanding at all. In short, the best rule for a jury to guide themselves by in such a case, is to judge whether the signs of madness, that are now pretended, would avail to acquit the same person of murdering another man; if not, there is no reason why they should be urged as a plea for acquitting him of murdering himself. But this is a little wide from my subject. However, it may be of use to show what little heed is to be given to a coroner's warrant, and that there is no reason, because a coroner prostitutes his oath, that the clergy should be so complaisant as to prostitute their office.'

"Under all the circumstances of the case, then, and in the absence of any proof that the rubric has been set aside by any subsequent and equal authority, we cannot see that the clergyman in the present instance acted illegally in refusing to read the Burial Service over the deceased. If, however, all the allegations of the petition presented are true, he went beyond this, and refused to allow the deceased to be buried at all in the churchyard, even without any service; and this we believe to be decidedly illegal, and certainly of questionable propriety; but the statements are as yet *ex parte*. . . ."

We must content ourselves with recording this expression of opinion.

If we were disposed to rest satisfied with a rough guess, from newspaper reports, of the state of serious crime in the kingdom, we might be induced to conclude that it was on the increase, so numerous of late have been the accounts of bloody and brutal murders and assaults. Our chief authority on these matters, however, Mr. Samuel Redgrave, of the Home-office, in his report on Judicial Statistics for 1858, gives us the following gratifying and hopeful information on the decrease of graver crimes :—

"The offences prosecuted in 1858 prove generally a decrease of the gravest crimes, both in the offences against the person, Class 1, and in a very marked degree in the violent offences against property, Class 2. The same may be said of the most serious offences comprised in Class 3 (offences against property without violence). And the returns evidence a very general decrease of offences arising probably as well from the preventive action of the newly-enlarged police system as from the improvement of the classes from which criminals spring. The malicious offences (against property) in Class 4 are a corroboration of this view. Such offences result from the revengeful and misguided passions of an ignorant population, and present many difficulties to detection and punishment. Fifty years since, frame-breaking was an offence of frequent occurrence in the manufacturing towns, but has long since disappeared. Later, threshing-machine-breaking prevailed throughout the agricultural districts : it is no longer heard of. Incendiarism was at the same time an alarming crime : it has since lost the class character which it then possessed, though it still lingers in constantly diminishing amount. I mentioned last year the absence of all offences of a political character, and the remark still applies to the criminal records." (p. xvi.)

The following remarks from the *Times* (Sept. 10) upon the Judicial Statistics of the past year are of considerable interest, and they present an ingenious view of crime as a profession :—

"The Judicial Statistics just published contain, amid some encouraging symptoms, the usual facts relating to crime. Everything seems to show what we technically call crime to be not the offspring of society at large—the aggregate of a quantity of isolated offences committed by individuals here, there, and everywhere, of all classes of the community—but that it is the production of a class which may not be improperly called the criminal class—a professional class living upon crime, as our trades and professions live upon their respective occupations. People are apt to think, when they take up a criminal return, that every crime is the first offence of some fresh man, and that the return represents the lapses of the community at large, and is fed by a constant supply of victims of first temptations, who have hitherto been tolerably respectable men. But everything shows that this is a mistake. The report before us only gives about 25,000 out of the number of criminals of last year as persons openly and apparently 'without occupations'—overt members of the trade or profession of crime; but there can be no doubt that this list extends really much further, and that crime is the profession of thousands who maintain the show of some regular calling, the overt sample showing only the real composition of the whole class. It has been calculated, indeed, that a good deal more than 100,000 persons live by crime in this country.

"It may naturally be asked how we are to account for this most gloomy and disastrous of all professions under the sun being the choice of thousands who have abundant talents, bodily and mental, to earn a prosperous subsistence by some regular calling. This class is by no means deficient in body or mind. A great deal of cleverness is required for crime, much more than for many a more profitable occupation. A burglar must be not only a bold and active, but a clever fellow. He has to choose his house, in the first place, with reference to various tests of advantageousness—nature of the household and establishment, vicinity of police, look of walls, doors, and windows, facilities for escape, &c. Thus crime requires what is a rare gift—the sudden concentration of mind, the power of urging it for a short time to the greatest quickness, vigilance, self-possession, decision. The commonest pickpocket, if he falters, is lost. Your good, honest labourer can get on very well with his wits always at an average height, which they never exceed by any chance; but the criminal must not go by clockwork, or let his mind operate quietly and regularly by the laws of nature—the happy privilege of many a good fellow who earns twenty or thirty shillings a week. It is quite true that many who enter the profession are not clever enough for it, and get caught soon, as a punishment for their presumption. Nor can the cleverest surmount all the chances—he gets caught at last; but there can be no doubt that there is a great deal of cleverness actually at work, and successfully at work—according to a certain standard of success—in the profession of crime.

"How, then, is it that these clever fellows—born, one would think, from their peculiar gifts, to shine in the world—select such a calamitous calling as that of crime, which, after terrors, alarms, and hair-breadth escapes innumerable, is almost sure to land them in penal servitude? All we can say in the matter is, that there is no accounting for tastes. It seems to be an ascertained fact that some people will do anything rather than do regular work. Regular work, so far from being distasteful to the majority of mankind, is, happily, the pleasure of life; they would not know what to do with themselves without it; they would be quite lost; even where there is a preliminary falter at starting, it is got over immediately. But some natures certainly show a remarkable repugnance for regular work—a repugnance which cannot be mistaken, but is a decided phenomenon of mind which we must recognise. It is difficult to analyze this condition of mind: it is quite different from that of common indolence, which is only a lazy *way of working*. We can no more get to the bottom of it than we can to the shying of a horse, or any other mysterious affection of animal nature. Men who have it look upon regular work as a mystery of which they do not know the end—a dreadful secret and nightmare. Upon the slightest actual entrance upon it they have sensations of despair, as if they were locked up in a dark room to wrestle with some strong monster. They are oppressed with the idea of an interminable struggle, which they do not see their way out of. They shrink from the abyss, as if they would never come out of it again in this hemisphere. It is this sort of blind—nervous, animal repugnance to steady employment which, in a large proportion of cases, makes the criminal. He will do anything rather than dig potatoes for a week running. He will exert himself, if he can do so, by fits and starts, in obedience to some strong momentary impulse; he will watch an opportunity eagerly; when the prey is coming he will listen breathlessly for a sound, and strain himself to apprehend quickly every circumstance; but there must be the sharp stimulus of cunning, the hurry of excitement to bring him out.

"Many of our criminals would not have been criminals at all if they had been born Red Indians. They would not have had to work. Labour, regular labour, is the trial of civilization, the criterion by which society now tests its respectable poor. There are many, however, who appear, unluckily for themselves, to be born for a different world or age of the world than that in which they live; there are wild, headstrong aristocrats, who spend their lives in a rebellion against society, but who would have made very good crusaders; and there are many criminals who would have made respectable Mohawks. We do not presume to enter into the mystery of Providence, which brings about this apparent incongruity, nor do we wish, of course, to protect anybody against the responsibilities of life. A man who lives in the 19th century must have the trials of civilization—not the trials of barbarism—and it is doubtless his own fault if he does not sustain them. His blind, animal shudder at the providential trial of labour is a sensation which may be got over, and we have no doubt that thousands of persons who have had it have got over it by simply going to work in spite of it. This has dispelled the evil charm and set them free; they have found what a mere dream of the imagination their previous idea of labour was. But the selfish, undisciplined nature, that never will do anything the least disagreeable to it—the rebel, the brute, the slave of impulse who disowns his conscience, are overpowered by the gloomy and odious nightmare; they are fairly taken captives, and they sink deeper and deeper into the moral impotence to which it consigns them. They take refuge in a life of crime, as the only alternative left by which to gain a subsistence, and the more they habituate themselves to the guilty stimulants of that life, the more revolting does a life of steady labour appear. Their conviction brings an additional difficulty—character is gone, nobody will employ them; and the criminal life—as a case in our police courts singularly illustrated the other day—which was a choice before, is almost a necessity after. Thus, in the case to which we refer, you have two criminals actually entreating the judge to transport them, as the only way of giving them a chance of recovery. 'What will be the good of our doing six years on the home stations, and then have no chance but to do the same thing again!' The criminal class thus gets more and more committed to its original choice, and crime grows more and more into a trade and profession, the regular occupation of life."

We cannot close our Retrospect without directing attention to the statements which have recently appeared in the *Times* concerning the abominable state of the solitary lunatic asylum which exists in Jamaica, and the wretched condition of its inmates, as well as the general mismanagement of lunacy affairs in the island. In a letter to the journal just named, a correspondent who signs himself "B," writes thus:—

"In Jamaica there is the most distinct evidence that the insane members of its population are grossly neglected and cruelly treated. Their wretched condition is even admitted in the Colonial Assembly; but, though it has called forth propositions to investigate and to remedy it, yet the petty differences of the island politicians, the plea of limited resources, and the prevalence of jobbery have thwarted every plan, and perpetuated the acknowledged grievance.

"There is but one institution professing to be a place of treatment and a refuge for the mentally disordered residents of the island. It is situated at Kingston, where it forms a section of the Public Hospital, and is under the same medical and general government with it. Its position is hygienically bad; it is within the town, overlooked by neighbouring streets, and enclosed within a high wall—conditions, one and all, condemnatory of its use as an asylum. Its structure has certainly the merit of simplicity. Two parallel one-storied buildings, each consisting of a row of 12 cells, constitute the institution; a wooden fence placed between the two rows is supposed to separate the male occupants of the one from the female of the other. At least, such was the æsthetical idea of the managers for the time being; but in practice it is frustrated by the lodging of female lunatics by night in some of the cells on the male side.

"As lunatics will multiply by accumulation in the course of years, it so happens that the 24 cells provided half a century ago for the accommodation of the lunatics in the island at that time, and under the influence of the views of treatment then in vogue, are utterly unfit and insufficient for present wants. Within those 24 cells—some 13 feet by 10 feet in area each—120 or more patients have been habitually crammed. Nay, the crowding is worse than this statement represents, for we have to deduct three, four, or more cells occupied by violent or by single patients of the more respectable class of society from the accommodation available for the 120 poor unfortunate creatures condemned to this vile prison-house. The consequence is that from 3 to 13 human beings, the victims of mental derangement, are crowded together in a room of the dimensions intimated, or thereabouts, locked in by night and left to themselves. A small aperture or window, which can be closed by a shutter, serves as the sole means of ventilation. An official report tells us what quantity of air is supplied to each occupant. It varies from 214 to 497 cubic feet; but the larger modicum is partaken by few. What must be the lot of a dozen poor wretches, mostly of the coloured races, shut up in a cell within the tropics with only 200 cubic feet of air each for respiration—air, too, most imperfectly renewed and laden with human exhalations? What it must be can be but indifferently conceived, yet its wretchedness is aggravated by every concomitant of misery. Bedsteads and bedding are provided only for a portion of the patients in the building; the rest have to repose on a fixed inclined wooden shelf, or on the stone floor of the cell, stripped of their clothes, and without even a mat beneath them. Add to this that they are virtually unwatched during the night, for the presence of a woman, called a nurse, lodged in one of the cells of the female division, and the occasional appearance at the outer gate of the establishment of one of two watchmen, having the general security of the hospital and asylum intrusted to them, along with duties as attendants on the male patients in the former, are worthless for the purposes of supervision.

"Who can tell what may pass within the narrow limits of this place of confinement, miscalled an asylum, during the night among the groups of lunatics—some vicious, some desponding, others violent, shut out from inspection, and well nigh out of hearing? Who could express astonishment on learning of fierce quarrels and of injuries among them? Or who could be surprised at learning that in the densely-crammed building, with its adjuncts, high enclosing walls, large cesspools, and open drains, diarrhœa and dysentery are constant visitants of the population?

"It is impossible within the limits of a letter to go into details, whether to finish the picture of the establishment by night, or to portray its aspect by day. Suffice it to say, the latter is the counterpart of the former; that classification is impossible in the building, and that medical and moral treatment are totally out of the question. Such a condition of the insane, in any region where civilization is found, in however feeble a degree, is scarcely supposable. Were it not a fact beyond dispute, one would hesitate to credit its existence in an English settlement, and still more so in one so long administered under the British Crown, and now boasting its Free Constitution, its Elective Assembly, and Legislative Council. It is a disgrace, a blot upon the civilization of the people of Jamaica, allied as they are to the British nation.

"It would have been more marvellous than the state of things sketched, had not its evils been to some extent recognised in the island. I have already remarked that this has been the case. So long ago as 1843, the proposition to erect a new and fitting asylum was entertained, and after elaborate inquiries, the grant of money by the Legislature, and the reception of a plan from England, the new edifice was commenced in 1847. At the end of 1851, however, so leisurely was the progress, less than one-third of the building intended for only 300 patients had been erected, although little short of 21,000*l.* had been expended, besides convict labour of the estimated value of 10,000*l.* In other words, mere living space for 100 patients, within a building without fittings or furniture, was all that was obtained at the extravagant outlay of 300*l.* per head.

"Having achieved thus much, the local authorities appear to have taken fright at the proposition to build for the insane population of their island, for they stayed further operations, and delivered over to the owls and bats the work already completed. In fact, except for a few weeks, during the ravages of cholera in Jamaica in 1851 and 1854, the unfinished new asylum has been uninhabited between eight and nine years."

The English Commissioners in Lunacy have endeavoured to rouse the Home Government to action upon this subject, but, it would appear, according to another correspondent of the *Times* (Sept. 8), with only slight success, for—

" . . . Although Sir E. B. Lytton, as Colonial Minister, stated in the House of Commons that it was not the intention of the office to send out any commission, he subsequently, in answer to the suggestions of the Commissioners in Lunacy, admitted the necessity of such a commission; and that since his Grace the Duke of Newcastle took office, he (the Duke) has expressed the strongest, though non-official, opinions to the same effect. I know all this, but the old complaint, 'circumlocution,' presents its slough-like expanse to retard the progress of immediate action. The Colonial-office still insists that the Governor and the House of Assembly of Jamaica (which, by the way, will not meet till the end of October) must again be consulted on the subject."

